# Electromagnetic enhancement of ordered silver nanorod arrays evaluated by discrete dipole approximation

**DOI:** 10.3762/bjnano.6.69

**Published:** 2015-03-09

**Authors:** Guoke Wei, Jinliang Wang, Yu Chen

**Affiliations:** 1 Department of Physics, Beihang University, Beijing 100191, China; 2 Photophysics Group, Centre for Molecular Nanometrology, Department of Physics, SUPA, University of Strathclyde, John Anderson Building, 107 Rottenrow, Glasgow G4 0NG, UK

**Keywords:** discrete dipole approximation (DDA), enhancement factor, near-field, silver nanorod array, surface-enhanced Raman scattering (SERS)

## Abstract

The enhancement factor (EF) of surface-enhanced Raman scattering (SERS) from two-dimensional (2D) hexagonal silver nanorod (AgNR) arrays were investigated in terms of electromagnetic (EM) mechanism by using the discrete dipole approximation (DDA) method. The dependence of EF on several parameters, i.e., structure, length, excitation wavelength, incident angle and polarization, and gap size has been investigated. “Hotspots” were found distributed in the gaps between adjacent nanorods. Simulations of AgNR arrays of different lengths revealed that increasing the rod length from 374 to 937 nm (aspect ratio from 2.0 to 5.0) generated more “hotspots” but not necessarily increased EF under both 514 and 532 nm excitation. A narrow lateral gap (in the incident plane) was found to result in strong EF, while the dependence of EF on the diagonal gap (out of the incident plane) showed an oscillating behavior. The EF of the array was highly dependent on the angle and polarization of the incident light. The structure of AgNR and the excitation wavelength were also found to affect the EF. The EF of random arrays was stronger than that of an ordered one with the same average gap of 21 nm, which could be explained by the exponential dependence of EF on the lateral gap size. Our results also suggested that absorption rather than extinction or scattering could be a good indicator of EM enhancement. It is expected that the understanding of the dependence of local field enhancement on the structure of the nanoarrays and incident excitations will shine light on the optimal design of efficient SERS substrates and improved performance.

## Introduction

Surface-enhanced Raman scattering (SERS) has attracted substantial interest over the past decades due to its potential applications in biological sensing and chemical analysis with molecular specificity and ultrahigh sensitivity, which can be even down to the level of single molecules [1–2]. In addition, SERS can be a label-free spectroscopic tool with capabilities in real-time and multi-component analysis. Previous studies showed that Raman signals from molecules adsorbed on nanostructured metal surfaces, especially noble metals (e.g., Ag, Au), could be amplified by a factor of about 10^6^ or even higher [3]. Although the underlying mechanism is still unclear, electromagnetic (EM) enhancement arising from the electric field in the vicinity of noble metal structure is considered as the dominant mechanism for such a dramatic Raman enhancement in most cases [4]. Both theoretical and experimental studies have revealed that the “hotspot”, which is the concentration of strong EM fields on nanometre-scale regions with high curvatures or gaps/junctions between closely packed nanoparticles, plays a significant role in SERS enhancements [5–6]. As recently demonstrated by Fang et al., a very small number of molecules residing at the hotspots can dominate the overall SERS signals [7]. Significantly, a single hotspot as small as 15 nm has been directly measured by single molecule imaging with accuracy down to 1.2 nm [8].

Tremendous efforts have been devoted to create efficient SERS substrates in recent years [9–11]. Among them, aligned Ag nanorod (AgNR) arrays fabricated by oblique angle deposition (OAD) were shown to be promising SERS substrates with enhancement factors of approximately 10^8^ [12–15]. However, the uniformity and reproducibility of SERS substrates remains a major challenge for the applications of SERS. Recently, it has been demonstrated that highly ordered Ag and Cu nanorod arrays can be fabricated by a guided OAD method, which may circumvent the problems of gap-size and diameter control, leading to the reproducible fabrication of highly SERS-active substrates [16].

The SERS enhancement not only depends on the intrinsic properties and the dielectric environment of the metal nanoparticles, but also on their shape, size and spatial arrangement. The incident wavelength, angle and polarization were also proven to greatly affect the performance of an SERS substrate. Previously, Chaney et al. observed that the SERS intensity was dramatically enhanced when the nanorod length increased from 190 to 508 nm in the random AgNR arrays prepared by OAD method. The high aspect ratio and the lateral overlap between adjacent nanorods were considered as the main factors responsible for this phenomenon [12]. Later studies demonstrated that there was an optimal length for the SERS enhancement in the OAD AgNR array [13]. A zig-zag AgNR structure that could generate hotspots at sharp corners also showed potential in enhancing the SERS performance [17]. So far, the understanding of the SERS mechanism in OAD AgNR arrays is still limited. In addition to EM mechanism, surface effect and anisotropic absorbance of molecules were proposed to interpret the SERS enhancement from the AgNR array substrate [18]. Limited systematic studies on OAD AgNR array structures and different measurement conditions used in experimental studies hindered the direct comparison.

Here, we took a systematic approach to investigate the SERS enhancements of the two-dimensional (2D) AgNR arrays from the perspective of EM enhancement mechanism by using the discrete dipole approximation (DDA) method [19]. We expect that the understanding of the dependence of local field enhancement on the structure of the nanoarrays and incident excitations will shine light on the optimal design of efficient SERS substrates and facilitate their applications in biomedical sensing and chemical analysis.

## Numerical calculations

### DDA method

DDA is a powerful and flexible method for describing the far-field and near-field properties of targets with arbitrary geometries in a complex dielectric environment [19–21]. In DDA, the continuum target is represented by a finite cubic array of polarizable point dipoles, which is excited by an applied EM field. Each dipole interacts with both of the external field and the induced electric fields generated by all other dipoles in this array. The response of this array to the incident light is then solved self-consistently by using Maxwell’s equations. Recently, an extension of DDA to periodic structures has been developed, allowing for the calculation of the optical properties of 1D and 2D arrays. The theoretical principle of the DDA for periodic targets has been described in more detail elsewhere [22]. Briefly, a “target unit cell” (TUC), repeated in single or double directions, is utilized to assemble the periodic array. In this case, each dipole interacts with the incident electric field and the electric fields scattered by all of the other dipoles in the TUC and the replicas of the TUC. The EM problem is then solved self-consistently through Maxwell’s equations. In a recent work, Kim et al. showed that this generalized DDA method was an efficient and versatile numerical approach for calculations of optical properties of AgNR array [23].

To investigate the SERS enhancement of AgNR arrays fabricated by OAD method in terms of EM mechanism, we simulated the local field enhancement of the nanoarrays in vacuum employing the open-source code DDSCAT 7.2 developed by Draine and Flatau [19], which has the capability of performing efficient “near-field” calculations in and around the target by using fast-Fourier transform (FFT) methods [21]. The cubic grid spacing was 3 nm in all calculations. The dielectric constants of Ag were obtained from the experimental data of Johnson and Christy [24]. The value of the interaction cut-off parameter γ was taken to be 0.01.

### Electromagnetic enhancement factor

The electromagnetic enhancement factor (EF) is commonly approximated by the following formula [25]:

[1]
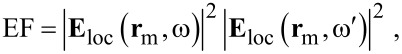


where **r**_m_ is the location of the molecule, **E**_loc_ is the enhancement of the local electric field (the ratio of the local field to the excitation field associated with the incident plane wave), and ω and ω′ are the incident and Stokes shifted frequencies, respectively. Normally, the shift is small and can be neglected compared to the plasmonic resonance width in metal nanosystems, leading to a fourth-power dependence [26],

[2]
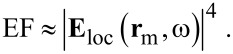


To evaluate the EF of SERS for the nanostructures, we calculated the sum and the average of the EF within a unit cell of the periodic lattice over the available surface area except the bottom, which was connected with the supporting substrate, by using EF_sum_ = ∫|**E**_loc_|^4^ *dS* and EF_avg_ = ∫|**E**_loc_|^4^ *dS*/∫*dS*, respectively [27]. Note that the value of |**E**_loc_| was not calculated exactly at the particle surface, but half a grid point (i.e., 1.5 nm) away from each exposed cube surface.

### Models

**Figure 1:**
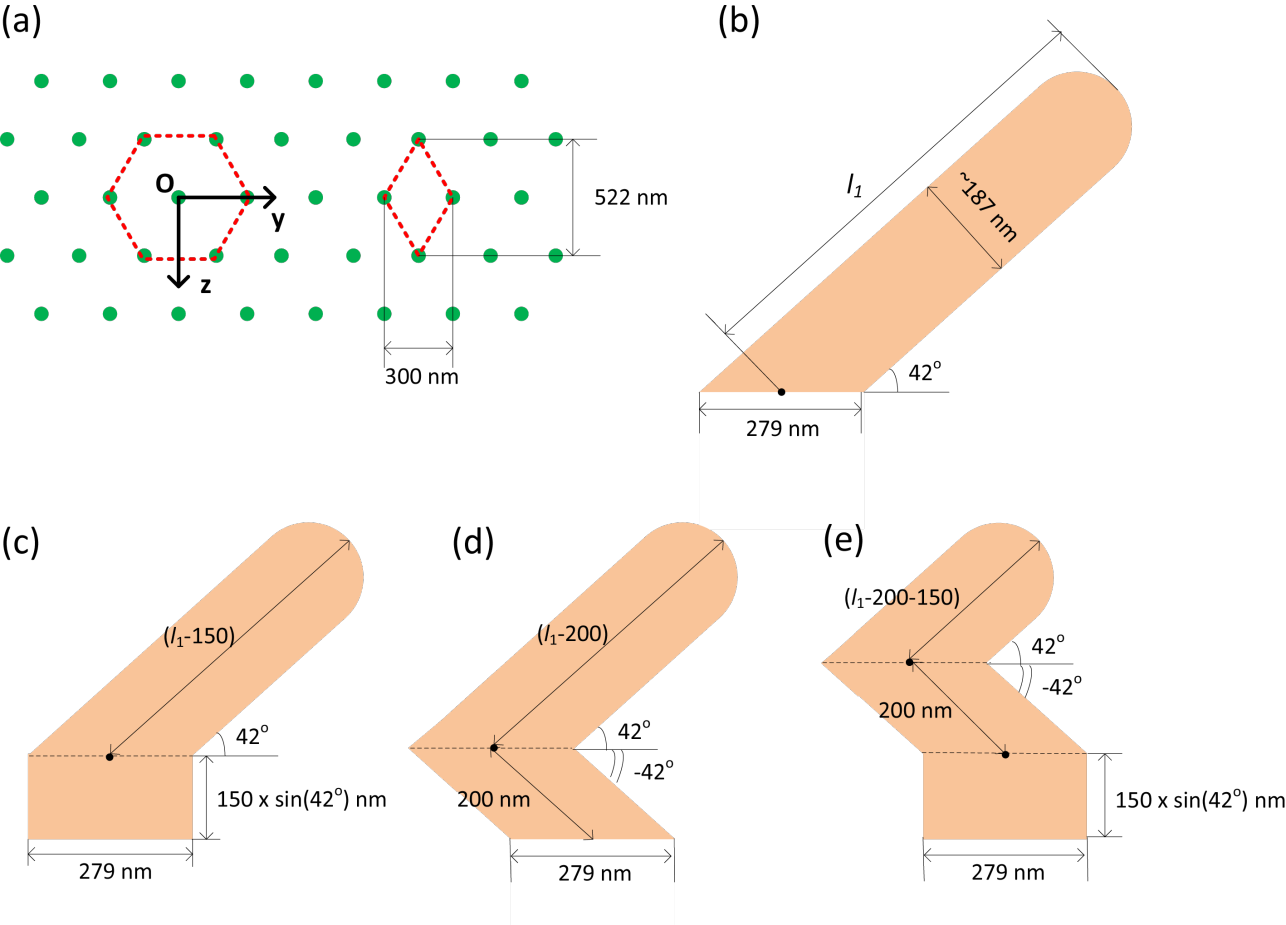
Schematic of the hexagonal pattern substrate (a) and four different target units: (b) S42; (c) S0:42; (d) S−42:42; (e) S0:−42:42.

The models used here are similar to those published previously in [16]. Figure 1 illustrates the regular hexagonal pattern substrate and four different target units considered in the calculations with the parameters shown on the schematic, selected from possible nanorod array structures fabricated by the guided OAD method [16]. The nanorods were arranged in the hexagonal lattice with a centre-to-centre distance of 300 nm unless otherwise noted (Figure 1a). The orientation of the oblique nanorods was chosen to be along the *y*-direction, and the tilting angle was set to 42° relative to the *y*-direction [16]. The upper oblique parts of the nanorods were all modelled as tilted cylinders with a hemispherical cap at each end, in order to avoid the “lightening rod effect” at the top edges of the nanorods in the electrodynamics simulations. The gaps between adjacent nanorods along the *y*-direction were fixed to 21 nm unless specified otherwise, resulting in a cylinder with a diameter of 187 nm. When investigating the effect of different structures on the SERS enhancement, the volume of each target unit was kept constant. This was achieved by considering a factor of sin(42°) when designing the height of the vertical pillar base in S0:42 and S0:−42:42. The nominal aspect ratio (AR), defined as *l*_1_/(187 nm), was used for all structures. For simplicity, the supporting substrates of the arrays were not considered in the simulations. Only the 2D AgNR array of S42 with AR = 3.5 and the excitation wavelength of 632.8 nm were investigated except in the sections of structure dependence and excitation-wavelength dependence.

## Results and Discussion

### Extinction for isolated nanorods and nanorod arrays

Typically, metal nanoparticle with anisotropic structure shows multiple plasmon resonances associated with different modes under appropriate excitations [20]. For AgNRs much smaller than the wavelength of light, the extinction spectra usually exhibit a transverse mode centred at around 420 nm and a longitudinal mode in the range of 500–1100 nm depending on the AR [28–29]. These are considered to arise from the dipole plasmon resonances. For AgNRs of large sizes, however, higher order modes of plasmon resonances can be excited [20]. As the target units investigated in the arrays consist of tilted rods, it is expected that both transverse and longitudinal modes can be excited when they are illuminated under normal incidence. Here, the normal incidence is defined as the light with the propagation direction parallel to the surface normal of the substrate (perpendicular to the *y*-direction).

**Figure 2:**
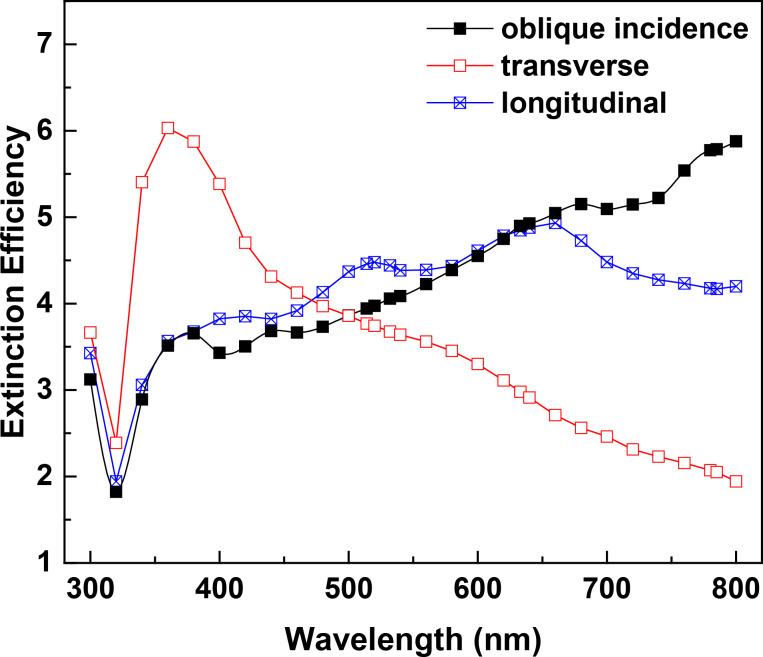
Extinction efficiency spectra of isolated S42 AgNR with AR = 3.5. The black curve corresponds to the situation in which the AgNR has a tilting angle of 42° and the propagation direction of the p-polarized light is parallel to the substrate surface normal. The red and blue curves represent the extinction efficiency of the target under the excitation of s-polarization and p-polarization, respectively, when the propagation direction of the light is perpendicular to the long axis of the nanorod.

Figure 2 shows typical extinction efficiency spectra of an isolated S42 AgNR of AR 3.5. Under normal incidence of p-polarization, the extinction spectrum has a broad band starting from 320 nm. A general trend of slow increase in the efficiency is apparent in the range of 400–800 nm, with some noticeable features at around 380, 440 and 680 nm. In order to identify the plasmon modes, the extinction efficiency spectra of the target under the s-polarized and the p-polarized excitations are also depicted in Figure 2, in which the propagation direction of the light is perpendicular to the long axis of the nanorod. A major plasmon resonance peak centred at 360 nm is found under the excitation of s-polarization, along with a broad shoulder at around 550 nm. These resonances can be assigned as dipole (550 nm) and quadrupole (360 nm) plasmon modes, respectively, as found in Ag nanoparticles of large sizes [20]. In the case of p-polarized excitation, the extinction spectra has a broad band ranging from 320 to 800 nm, with three distinguishable peaks located at around 400, 520 and 660 nm. Generally, the number of plasmon modes increases with the increasing of asymmetry. The resonance at 660 nm is ascribed to the dipole plasmon mode, while the resonances at 520 nm and 400 nm may be related to higher-order multipolar plasmon modes. Obviously, the extinction efficiency spectra of the tilted target unit under the normal incidence of p-polarization consist of both transverse and longitudinal modes.

**Figure 3:**
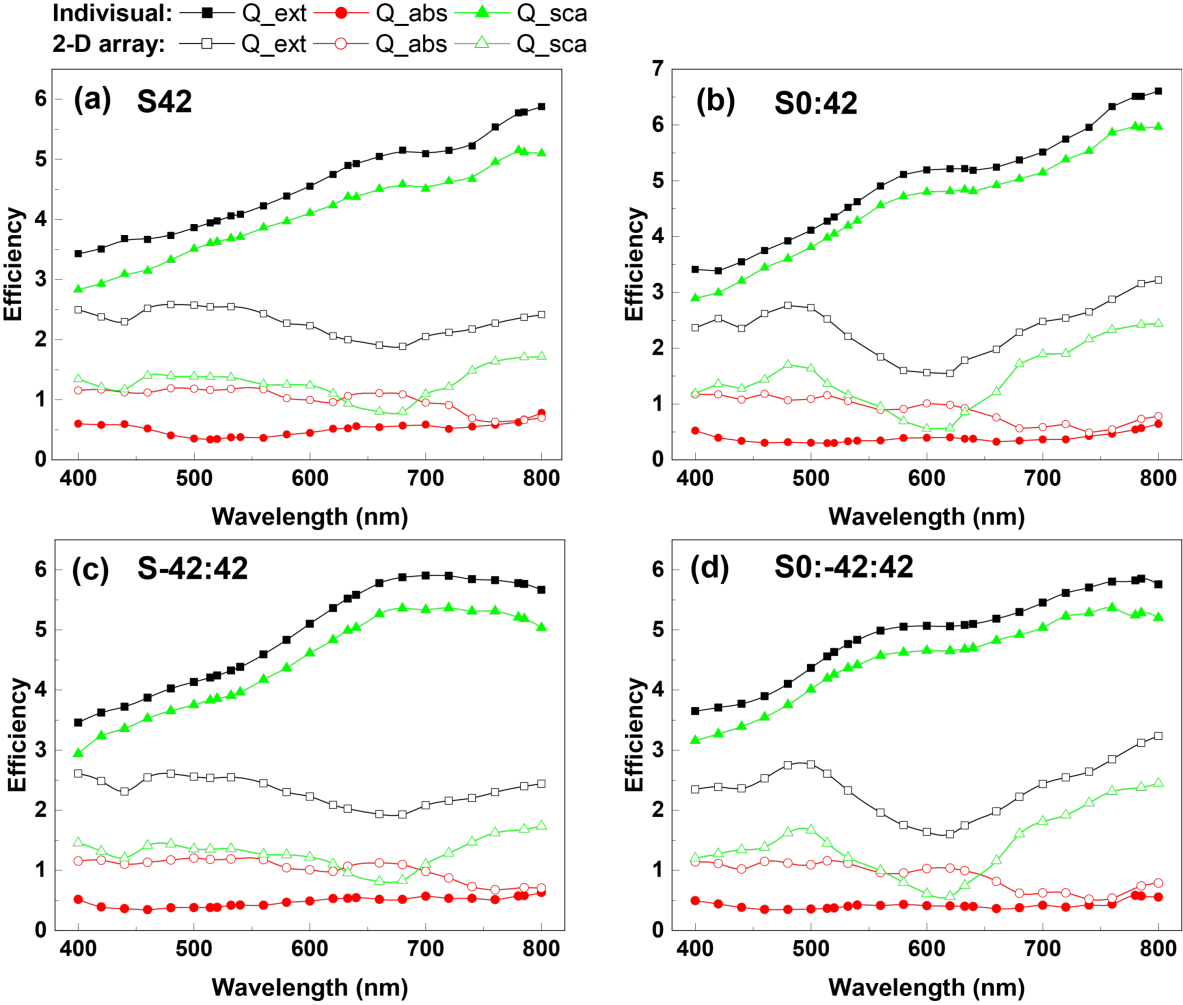
Extinction, absorption and scattering efficiencies of the four target units with AR = 3.5 and their 2D hexagonal arrays with a periodicity of 300 nm: (a) S42, (b) S0:42, (c) S−42:42, (d) S0:−42:42. The light is incident along the substrate surface normal with p-polarization.

As the target units form 2D arrays, the optical properties change due to the coupling effect between neighbouring rods, as depicted in Figure 3. Interestingly, the extinction spectra of the S42 and the S−42:42 arrays are almost the same, so are those of the S0:42 and the S0:−42:42 arrays, although the optical spectra of corresponding individual target units are different from each other. This is probably due to the strong coupling effect resulted from the narrow gap between nanorods investigated here. Significant increases of absorption efficiency are found for all four arrays, (almost doubled in the ranges of 400–700 nm for S42/S−42:42, and 400–640 nm for S0:42/S0:−42:42 arrays), in comparison to that of individual target units. Contributions from absorption and scattering are comparable in these ranges. At long wavelengths, (740 nm for S42/S−42:42; 700 nm for S0:42/S0:−42:42 arrays), absorption decreases and scattering dominates the extinction spectra. The dependence of the scattering on the wavelength shows clear oscillations, different from that of individual target units. Moreover, the scattering efficiencies are significantly reduced, resulting in much decreased extinction efficiencies in comparison with that of individual targets.

### Effects of structure and length

**Figure 4:**
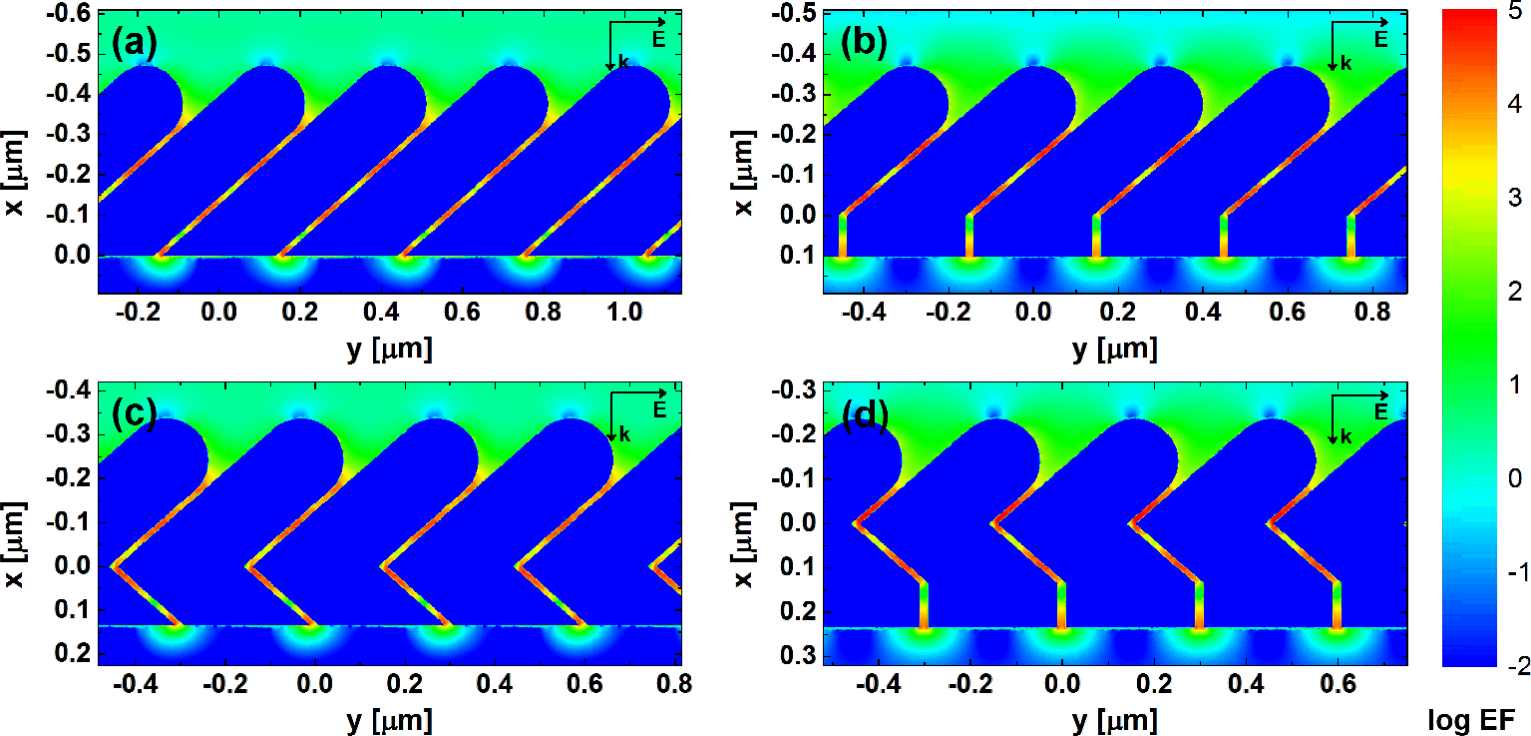
EF distributions obtained from DDA calculations for AgNR 2D hexagonal arrays of different structures with AR = 3.5: (a) S42, (b) S0:42, (c) S−42:42, (d) S0:−42:42. The colour bar is at log scale. The excitation wavelength is 632.8 nm and the polarization is parallel to the *y*-direction. All internal fields are set to 0.01 for visual clarity.

As shown in the previous section, both transverse and longitudinal modes in the tilted nanorods can be excited simultaneously by the p-polarized light under normal incidence. The coupling of EM fields of neighboring rods greatly enhances the local fields, forming so-called “hotspots”. Figure 4 shows the calculated contours of EF for AgNR 2D hexagonal arrays of different structures with AR = 3.5. Multiple hotspots are found distributed in the gaps. However, the number and the intensity of the hotspots are structure-dependent. Both S42 and S−42:42 have four hotspots in each gap, while S0:42 and S0:−42:42 have three hotspots between adjacent nanorods. The brightest hotspots are found in S0:42 and S0:−42:42. Quantitative analysis shows that the average EFs of S42 and S−42:42 are comparable, which are 797 and 793, respectively, while S0:−42:42 shows the strongest EF_avg_ of 1228, followed by S0:42 with EF_avg_ = 1006. Corners/bends are usually considered to give rise intense fields for SERS due to the “lightening rod effect” [5–6,17]. However, similar EFs from S42 and S−42:42, as well as S0:−42:42 and S0:42, show that there are no significant contribution from near-field enhancement right at the corners/bends. This indicates that strong EM coupling in the narrow gap is the dominant factor for the near-field enhancement in these arrays.

**Figure 5:**
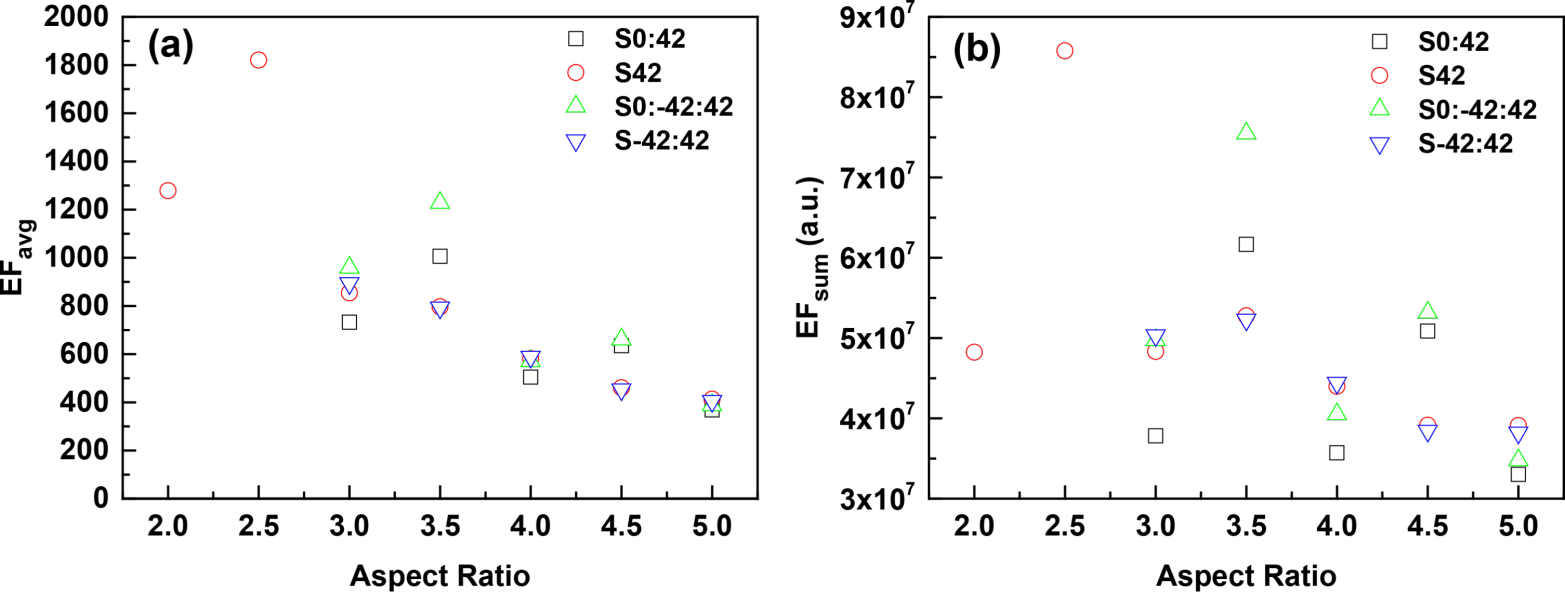
The average EFs (a) and the total EFs (b) of AgNR 2D hexagonal arrays with different structures and ARs. The excitation wavelength is 632.8 nm and the polarization is parallel to the *y*-direction.

We further investigated the dependence of EF on the length of AgNR in different structures. A range of aspect ratios from 2.0 to 5.0 was chosen for S42 arrays, while ARs ranging from 3.0 to 5.0 were applied to other structures due to the constraints of the structure parameters investigated in this work. The number of “hotspots” between adjacent nanorods was found to increase in all four structures as their ARs increased. The EF_avg_ and EF_sum_ of each structure with varying ARs are shown in Figure 5a and Figure 5b, respectively. It is interesting to find that the EFs of S0:42 and S0:−42:42 exhibit a similar behavior as the AR increases, both have a general decreasing trend but in an oscillating manner. The EF_avg_ of S42 reaches its maximum at AR = 2.5, more than four times than that in the case of AR = 5.0. It is worth noting that the EFs of S42 and S−42:42 are comparable at the same AR region between 3.0 and 5.0. And both of their EF_avg_ decrease as the AR increases, consistent with the simulation result from Cu nanorod arrays in our previous work [16]. As the increase of surface area can result in an increased amount of molecular adsorbate and in turn an enhanced SERS intensity, here we take the surface area effect into account and compare the total SERS enhancement (EF_sum_). As shown in Figure 5b, the surface effect is clearly visible at certain ARs and seems also depending on the structures of target units, although EF_sum_ shows a similar trend against AR as EF_avg_ does.

### Effect of the excitation wavelength

**Figure 6:**
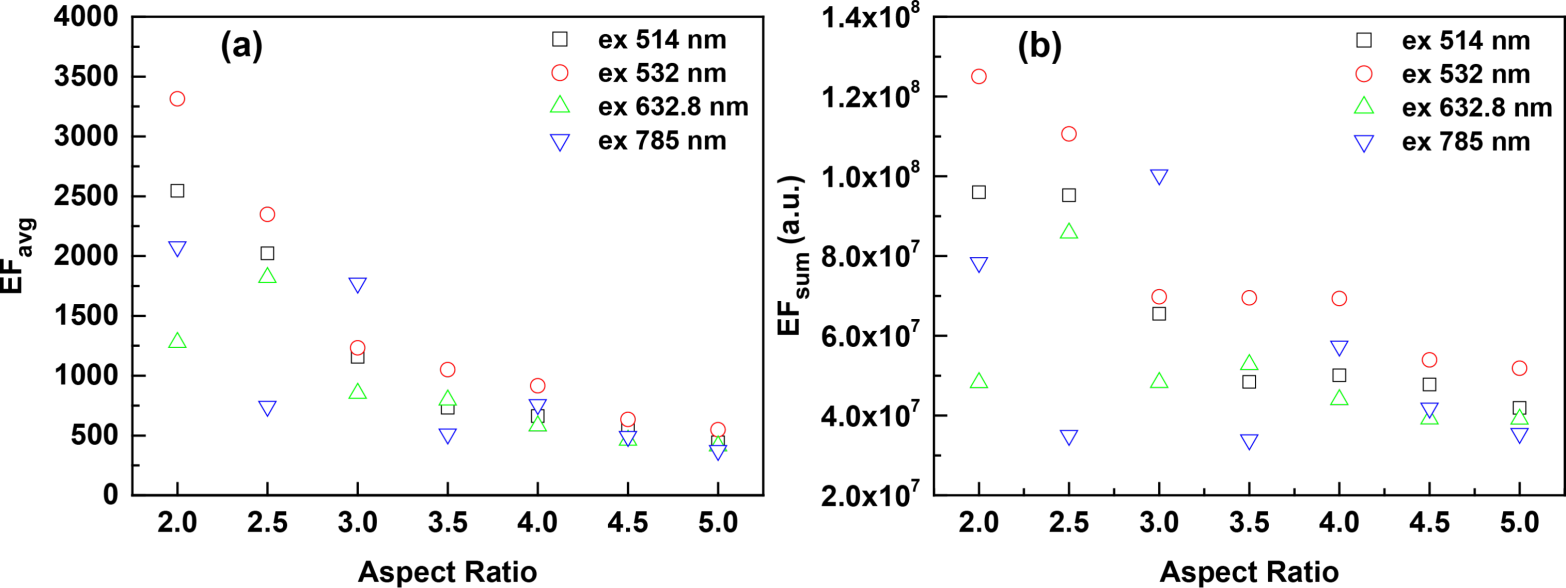
The average EFs (a) and the total EFs (b) of S42 AgNR 2D hexagonal arrays with different ARs, illuminated by different excitation wavelengths (i.e., 514, 532, 632.8 and 785 nm) with the polarization parallel to the *y*-direction.

Since the SERS effect is a near-field phenomenon and related to the localized surface plasmon resonance (LSPR) of the nanostructures, it is expected to exhibit a behavior that depends on the excitation wavelength. Here, we calculated the EFs of the S42 AgNR arrays with the commonly used excitation wavelengths, i.e., 514, 532, 632.8 and 785 nm, as shown in Figure 6. As can be seen from Figure 6a, the excitation of 532 nm gives the most intense EF_avg_ at each AR except AR = 3.0. The EF_avg_ of the AR = 2.0 array illuminated by 532 nm is more than twice than that of 632.8 nm. It is interesting that the differences of EF_avg_ between different excitation wavelengths become insignificant at large ARs. The EF_avg_ decreases under both excitations of 514 and 532 nm as the AR increases, while the EF_avg_ shows an oscillating behavior at low ARs in the cases of 632.8 and 785 nm excitations. Notably, the array with AR = 2.0 excited by 532 nm exhibits the most intense EF_sum_ despite of its relative small surface area, as shown in Figure 6b.

**Figure 7:**
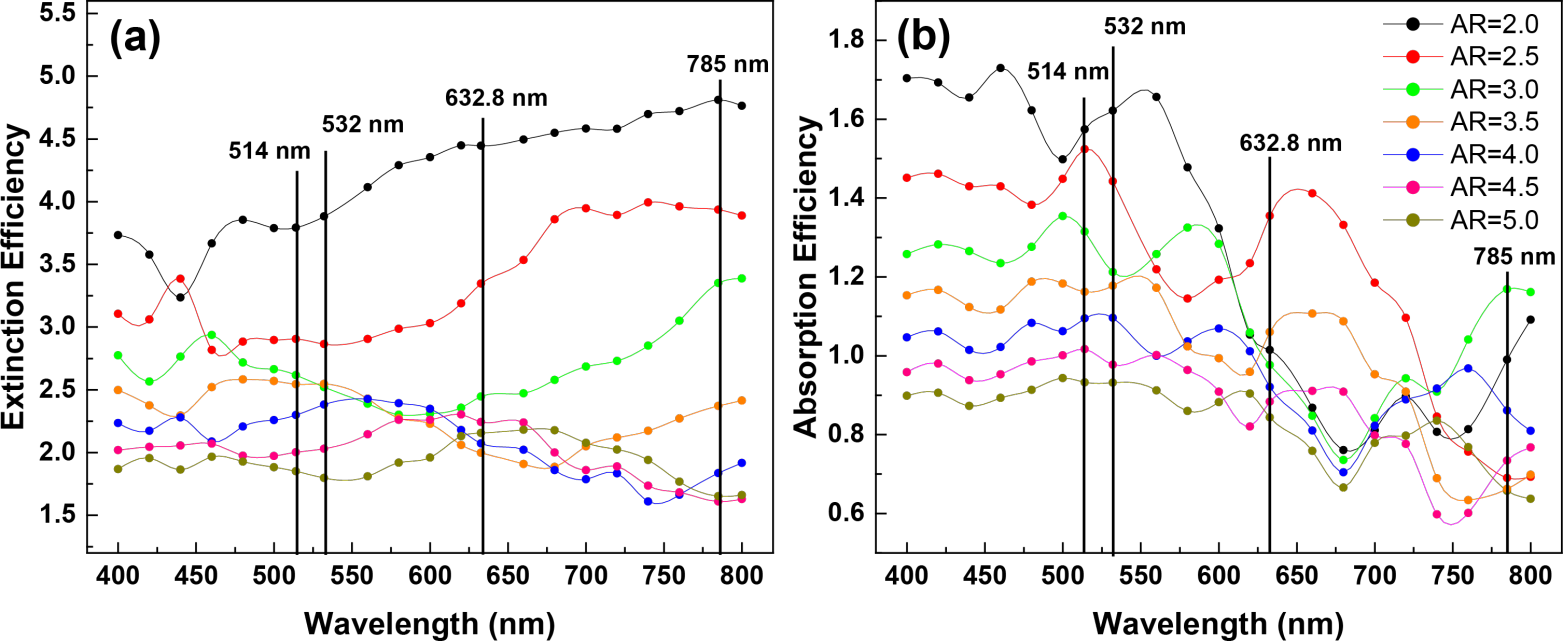
(a) Extinction efficiency spectra of S42 AgNR 2D hexagonal array with AR ranging from 2.0 to 5.0; (b) absorption efficiency spectra of S42 AgNR array with AR ranging from 2.0 to 5.0. The incident polarization is parallel to the *y*-direction. Vertical solid lines indicate the excitation laser wavelengths used in Figure 6.

In order to understand the wavelength dependence of the EM enhancement, the extinction and absorption efficiency spectra of S42 AgNR array with varying ARs were also calculated and are given in Figure 7. It is clear that there is no direct correlation between the extinction efficiency and the average EF or the total EF. However, the dependence of absorption efficiency on the AR at each excitation wavelength shows a similar trend as the total EF. Typical features, such as oscillating behavior at low ARs in the cases of 632.8 and 785 nm excitations and highest efficiency at AR = 2.0 under 532 nm excitation, are consistent with what was observed in Figure 6. It suggests that absorption efficiency could be used as an indicator for SERS enhancement. Nevertheless, the connection between the absorption/extinction spectra and the enhancement in SERS is still not fully understood [30] and requires further investigation.

### Effect of incident angle

**Figure 8:**
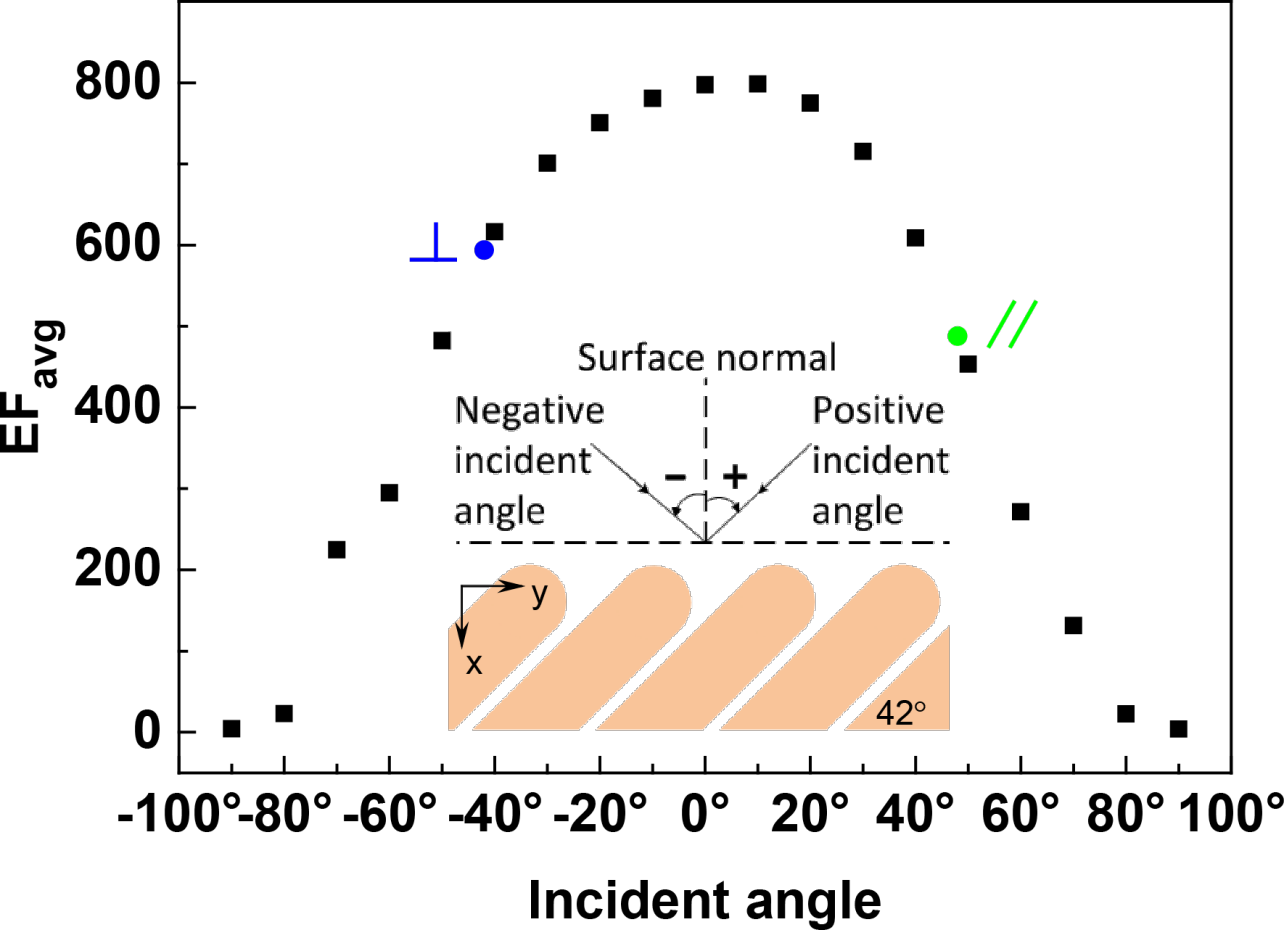
The angular dependent EF_avg_ of S42 AgNR 2D hexagonal array with AR = 3.5. The excitation wavelength is 632.8 nm. The insert illustrates the incident direction and angle. The incident polarization is in the *x*–*y* plane and perpendicular to the propagation. The symbols, 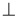 and //, denote that the incident directions are perpendicular and parallel to the long axis of the nanorods, respectively.

As is evident in Figure 8, the EF_avg_ strongly depends on the incident angle. The incident angle is defined as the angle with respect to the surface normal, as illustrated in the insert of Figure 8. The most intense EF_avg_ is obtained when the array is illuminated at a positive angle of about 10° (38° towards the long axis of nanorod). At this angle, the incident direction is neither parallel nor perpendicular to the long axis of the nanorods. The EF_avg_ decreases dramatically when the incident angle deviates from the optimum value. A similar asymmetric angular dependence of the SERS response was experimentally observed by Liu et al. in a tilted AgNR array with a tilting angle of ca. 17° [31], for which the maximum SERS intensity was obtained at an incident angle of about 45° off the surface normal. A modified Greenler’s model was also proposed to interpret this phenomenon. In this model, the molecule adsorbed on the side of the nanorod is treated as a dipole perpendicular to the long axis of the nanorod, while the surface of the nanorod was considered as a planar surface. The SERS intensity was assumed to be proportional to the mean square of total scattered field that was calculated by using classical electrodynamics. According to this model, the optimal incident angle increases as the tilting angle of nanorod (with respect to the surface normal) decreases [32]. Therefore, it is not surprising that the optimal incident angle found in our simulation is smaller than that reported in [31]. In fact, the angular dependence of near-field enhancement was also found in the vertical AgNR arrays. It has been revealed that different modes of surface plasmon resonance can only be excited by certain angles of incidence, leading to different near-field enhancements [23,33].

### Effect of incident polarization

**Figure 9:**
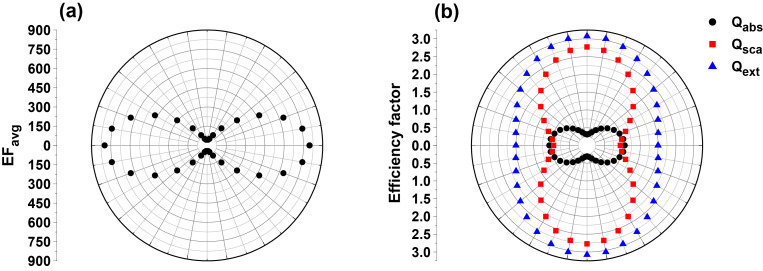
The polarization-dependent EF_avg_ (a) and the corresponding absorption (black), scattering (red) and extinction (blue) efficiency factors (b) of S42 AgNR 2D hexagonal array with AR = 3.5. The excitation wavelength is 632.8 nm.

Figure 9a shows the polarization dependence of EF_avg_ from S42 AgNR hexagonal array with AR = 3.5. The excitation wavelength is 632.8 nm, and the wave vector is perpendicular to the substrate. The polarization angle is defined as the angle between the electric-field vector and the *y*-axis as shown in Figure 1a. The most intense EF_avg_, 797, occurs at polarization angles of 0 and 180°. This is caused by the strongest EM coupling effect between adjacent nanorods when the exciting electric field vector is polarized along the interparticle axis (*y*-axis), as is well known in the particle dimer system [34–35]. The EF_avg_ of the array is quite sensitive to the polarization. As the polarization deviates from 0 and 180°, the EF_avg_ rapidly decreases, reaching a minimum value of 44 at polarization angles of 90 and 270°.

The polarization dependence of the optical cross sections corresponding to Figure 9a is shown in Figure 9b. The efficiency factors of absorption, scattering and extinction are defined as the ratios of the total cross sections for absorption, scattering and extinction per TUC to the geometrical cross-section of equal-volume sphere in one TUC, respectively [22]. It is found that the absorption shows a polarization with maxima at 0 and 180°, opposite to scattering and extinction that reach the maxima at polarization angles of 90 and 270° and the minima at 0 and 180°. Interestingly, the absorption follows the same polarization dependence as the EF_avg_, while the scattering and extinction exhibit a different behavior. Previously, Zhao et al. observed that the anisotropy of the SERS polarization was different from that of the polarized UV–vis absorbance of a nonplanar AgNR array substrate [36]. Practically, the UV–vis absorption spectrum measured in the experiment is the sum of absorption and scattering, i.e., extinction. So, the experimental observation is in line with this simulation result. The simulation result also suggests that the absorption rather than the extinction or scattering could be an indicator of EM enhancement in SERS performance, in line with the observation in Section “Effect of excitation wavelength”.

### Effect of lateral gap size

**Figure 10:**
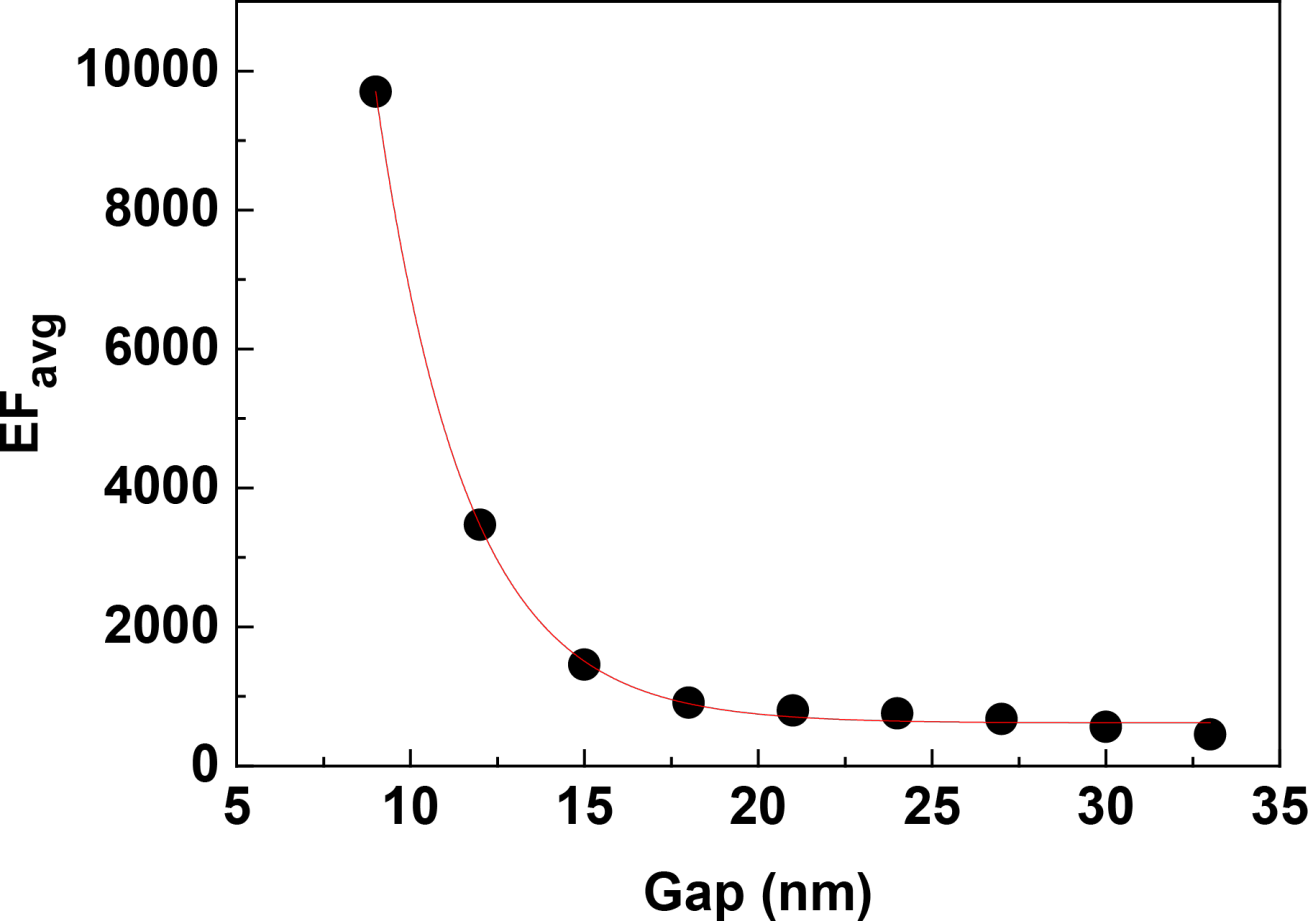
The dependence of EF_avg_ on the gap size along the *y*-direction in S42 AgNR 2D hexagonal array with AR = 3.5. The solid curve is an exponential fitting result. The excitation wavelength is 632.8 nm and the polarization is parallel to the *y*-direction.

EF_avg_ is highly sensitive to gap size, especially to small gap sizes below 15 nm, as shown in Figure 10. There is a dramatic decrease of EF_avg_ with the increase of gap size from 9 to 18 nm, and a much slower decrease with further increase of gap size. The gap size has been a crucial parameter of the SERS substrates because of the strong EM coupling effect at the nanometre scale [37–39]. Due to the challenges of fabricating ordered AgNR arrays by the OAD method, the effect of gap size in those arrays have not been experimentally investigated, yet. However, semi-ordered AgNR arrays were obtained by an OAD technique employing 2D Au nano-post arrays in square lattice as seed patterns [40]. The SERS intensities were shown to increase monotonically with the decreasing separation of AgNRs [40], which is consistent with our simulation results.

### Random vs ordered arrays

Although the tilted AgNR arrays fabricated by the OAD method were shown to have SERS enhancement factors greater than 10^8^, they were randomly distributed [12–13], which presents a challenge towards highly uniformed and reproducible SERS substrates. Hence, efforts have been devoted to produce well-patterned AgNR arrays [16,40]. It is interesting to compare the EFs of random and ordered arrays through theoretical simulations. However, due to the complexity of the 2D arrays, it is difficult to model a truly random AgNR array. Here, target units consisting of six AgNRs arranged in the *y*-direction with different gap sizes are used to model the 2D random arrays. The averages of the gap sizes in the target units are 21 nm, and the gap sizes between the target units along the *y*-direction are set to 21 nm, so that the average gap size along the *y*-direction is the same as that of the 2D ordered array. The gap sizes and the standard deviations (STDEVs) are shown in Table 1.

**Table 1:** Gap sizes and standard deviations in the target units used in modelling the AgNR 2D random arrays.

case	gap 1 (nm)	gap 2 (nm)	gap 3 (nm)	gap 4 (nm)	gap 5 (nm)	STDEV (nm)

1	21	24	18	27	15	4.7
2	21	30	12	24	18	6.7
3	21	30	12	27	15	7.6
4	21	24	18	33	9	8.7
5	21	27	15	33	9	9.5
6	21	30	12	33	9	10.6

**Figure 11:**
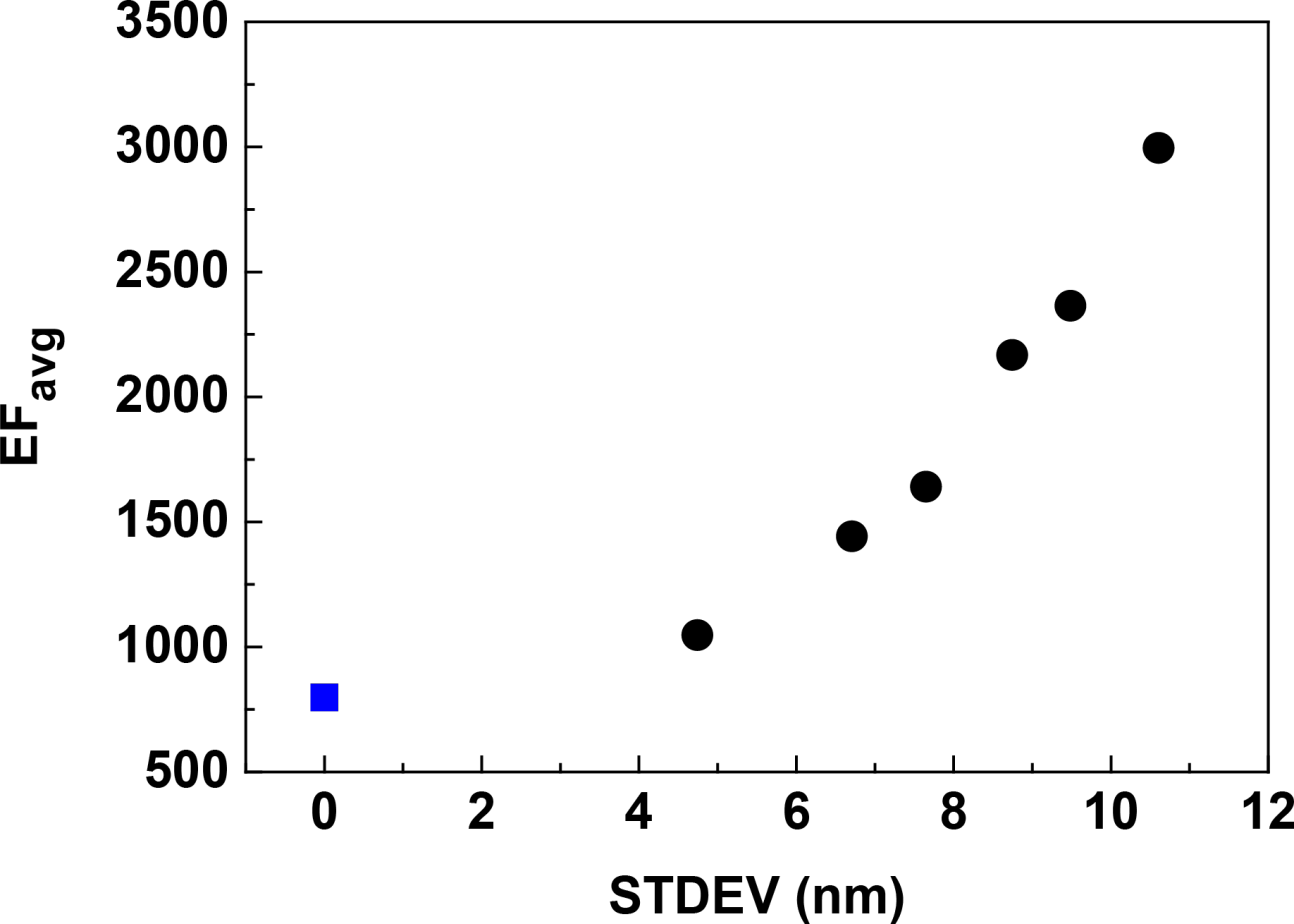
The dependence of EF_avg_ on the standard deviation of the gap size along the *y*-direction in the S42 AgNR 2D random array with AR = 3.5. The average gap size is 21 nm in the *y*-direction. The excitation wavelength is 632.8 nm and the polarization is parallel to the *y*-direction. The blue square in the figure indicates the ordered one with the gap size of 21 nm in the *y*-direction.

As shown in Figure 11, it is interesting that the EFs of the random arrays are all higher than that of the ordered array. Moreover, the EF increases monotonically as the STDEV of the gap size increases. Remarkably, the random array with a gap size STDEV of 10.6 nm shows a more than three times stronger EF than that of the ordered one. This indicates that the random arrays with the same average gap size (21 nm) as the ordered one can show a better SERS performance, which is consistent with the exponential dependence of the EF on the gap size, as demonstrated in Figure 10. This again manifests the significance of hotspots in defining total SERS intensity as revealed by Fang et al. experimentally [6]. It is worth pointing out that the difference of EF between random and ordered array is less significant when the average gap size is large and STDEV is small, because the gap sizes are then out of the region of rapidly changing EFs (Figure 10).

### Effect of diagonal periodicity

**Figure 12:**
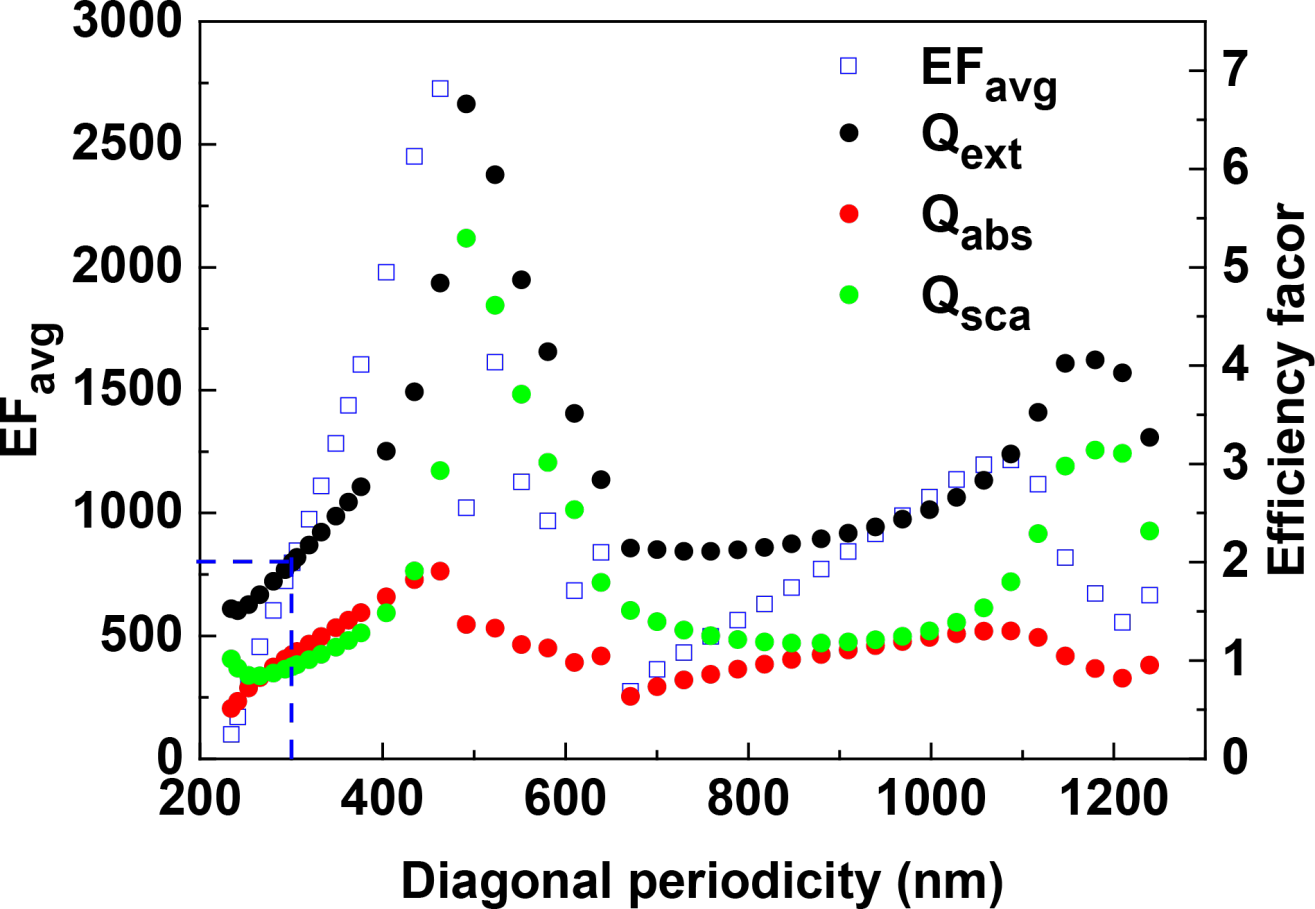
The dependence of EF_avg_ and extinction, absorption and scattering efficiency factors on the diagonal periodicity in the S42 AgNR 2D array with AR = 3.5. The excitation wavelength is 632.8 nm and the polarization is parallel to the *y*-direction. The dashed lines indicate the diagonal periodicity of the ordered array arranged in a regular hexagonal pattern.

We have shown that the variations of the gap size in the *y*-direction have a strong influence on the EF of SERS. However, the EF of a 2D array depends not only on the periodicity in the *y*-direction (denoted as lateral periodicity) but also on the periodicity in the diagonal directions. Here, we fixed the lateral periodicity to 300 nm, and investigated the dependence of EF on the diagonal periodicity. Figure 12 shows that a smaller diagonal periodicity, i.e., smaller gap size, does not necessarily result in stronger EF in the 2D arrays. In fact, the EF of the 2D array oscillates as the diagonal periodicity increases from 234 to 1239 nm (diagonal gap size varying from 21 to 1050 nm). The EF_avg_ of the ordered array arranged in a regular hexagonal pattern is more than three times lower than that of the ordered array with the diagonal periodicity of 463 nm. It is clear that diagonal periodicity plays an important role in the SERS enhancement for the 2D array but the dependence of EF on the diagonal gap is more complicated than that on the lateral gap and the mechanism needs further investigations. Nevertheless, this simulation indicates a new dimension to design OAD AgNR arrays for optimized SERS performance.

Also shown in Figure 12 are the absorption, scattering and extinction efficiency factors at the excitation wavelength of 632.8 nm. It clearly demonstrates that absorption follows a similar trend as the EF_avg_ does, different from extinction or scattering. This becomes more obvious for a diagonal periodicity larger than 600 nm. Recently, Near et al. found that the field strength within a plasmon mode trends with the absorption in a silver nanocube [41], which is in line with this simulation. This result is also consistent with our simulations on incident polarization effect (Figure 9), indicating that the absorption rather than the extinction or scattering may be a good indicator of the EM enhancement.

## Conclusion

The enhancement factor of SERS of 2D hexagonal silver nanorod arrays was investigated by using the discrete dipole approximation method. The computational studies clearly showed that “hotspots” were distributed in the gaps between adjacent nanorods, and the narrow gaps resulted in strong EFs. The excitation of 532 nm gives the most intense EF_avg_ at each AR except AR = 3.0, and the array with AR = 2.0 excited by 532 nm showed the most intense EF_sum_ despite of the smallest surface area. However, the influence of different excitation wavelengths on the EF became insignificant as the AR was over 4.0. The EF was found to be strongly dependent on the polarization of the incident light. The most intense EF was obtained when the array is illuminated with an incident angle about 10° off the surface normal. The simulations of AgNR arrays of different lengths revealed that increasing rod length generated more “hotspots” but not necessarily increased EF. The EM enhancement of 2D random AgNR arrays was compared with that of an ordered array of the same average gap size. It was found that the average EF of random arrays was stronger than that of an ordered one with the same average gap of 21 nm, which can be explained by the exponential dependence of the average EF on the lateral gap size. Although the narrow lateral gap results in strong EF, the dependence of EF on the diagonal gap shows an oscillating behavior, which implied that the SERS substrates could be optimized by adjusting the diagonal/longitudinal periodicity. The simulation results also indicated that absorption rather than extinction or scattering could be a good indicator of EM enhancement.

## References

[1] Nie S, Emory S R (1997). Science.

[2] Kneipp K, Wang Y, Kneipp H, Perelman L T, Itzkan I, Dasari R R, Feld M S (1997). Phys Rev Lett.

[3] Camden J P, Dieringer J A, Zhao J, Van Duyne R P (2008). Acc Chem Res.

[4] Moskovits M (1985). Rev Mod Phys.

[5] Moskovits M (2005). J Raman Spectrosc.

[6] Kleinman S L, Frontiera R R, Henry A-I, Dieringer J A, Van Duyne R P (2013). Phys Chem Chem Phys.

[7] Fang Y, Seong N-H, Dlott D D (2008). Science.

[8] Cang H, Labno A, Lu C, Yin X, Liu M, Gladden C, Liu Y, Zhang X (2011). Nature.

[9] Banholzer M J, Millstone J E, Qin L, Mirkin C A (2008). Chem Soc Rev.

[10] Wang Y, Schlücker S (2013). Analyst.

[11] Sharma B, Fernanda Cardinal M, Kleinman S L, Greeneltch N G, Frontiera R R, Blaber M G, Schatz G C, Van Duyne R P (2013). MRS Bull.

[12] Chaney S B, Shanmukh S, Dluhy R A, Zhao Y-P (2005). Appl Phys Lett.

[13] Driskell J D, Shanmukh S, Liu Y, Chaney S B, Tang X-J, Zhao Y-P, Dluhy R A (2008). J Phys Chem C.

[14] Liu Y-J, Chu H Y, Zhao Y-P (2010). J Phys Chem C.

[15] Song S, Keating M, Chen Y, Placido F (2012). Meas Sci Technol.

[16] Keating M, Song S, Wei G, Graham D, Chen Y, Placido F (2014). J Phys Chem C.

[17] Zhou Q, Zhang X, Huang Y, Li Z, Zhao Y, Zhang Z (2012). Appl Phys Lett.

[18] Liu Y-J, Zhang Z-Y, Zhao Q, Dluhy R A, Zhao Y-P (2009). J Phys Chem C.

[19] Draine B T, Flatau P J (1994). J Opt Soc Am A.

[20] Kelly K L, Coronado E, Zhao L L, Schatz G C (2003). J Phys Chem B.

[21] Flatau P J, Draine B T (2012). Opt Express.

[22] Draine B T, Flatau P J (2008). J Opt Soc Am A.

[23] Kim S, Jung Y, Gu G H, Suh J S, Park S M, Ryu S (2009). J Phys Chem C.

[24] Johnson P B, Christy R W (1972). Phys Rev B.

[25] Kerker M, Wang D-S, Chew H (1980). Appl Opt.

[26] Stockman M I, Kneipp K, Moskovits M, Kneipp H (2006). Electromagnetic Theory of SERS. Surface-Enhanced Raman Scattering – Physics and Applications.

[27] Li S, Pedano M L, Chang S-H, Mirkin C A, Schatz G C (2010). Nano Lett.

[28] Orendorff C J, Gearheart L, Jana N R, Murphy C J (2006). Phys Chem Chem Phys.

[29] Mahmoud M A, El-Sayed M A (2013). J Phys Chem Lett.

[30] Le Ru E C, Galloway C, Etchegoin P G (2006). Phys Chem Chem Phys.

[31] Liu Y, Fan J, Zhao Y-P, Shanmukh S, Dluhy R A (2006). Appl Phys Lett.

[32] Liu Y-J, Zhao Y-P (2008). Phys Rev B.

[33] Evans P R, Kullock R, Hendren W R, Atkinson R, Pollard R J, Eng L M (2008). Adv Funct Mater.

[34] Camden J P, Dieringer J A, Wang Y, Masiello D J, Marks L D, Schatz G C, Van Duyne R P (2008). J Am Chem Soc.

[35] Shegai T, Li Z, Dadosh T, Zhang Z, Xu H, Haran G (2008). Proc Natl Acad Sci U S A.

[36] Zhao Y-P, Chaney S B, Shanmukh S, Dluhy R A (2006). J Phys Chem B.

[37] Xu H, Bjerneld E J, Käll M, Börjesson L (1999). Phys Rev Lett.

[38] Zou S, Schatz G C (2005). Chem Phys Lett.

[39] Wustholz K L, Henry A-I, McMahon J M, Freeman R G, Valley N, Piotti M E, Natan M J, Schatz G C, Van Duyne R P (2010). J Am Chem Soc.

[40] Liu Y-J, Zhang Z-Y, Dluhy R A, Zhao Y-P (2010). J Raman Spectrosc.

[41] Near R, Hayden S, El-Sayed M (2012). J Phys Chem C.

